# Growth Hormone (GH) and Cardiovascular System

**DOI:** 10.3390/ijms19010290

**Published:** 2018-01-18

**Authors:** Diego Caicedo, Oscar Díaz, Pablo Devesa, Jesús Devesa

**Affiliations:** 1 Department of Angiology and Vascular Surgery, Complejo Hospitalario Universitario de Pontevedra, 36701 Pontevedra, Spain; diego.caicedo.valdes@sergas.es; 2 Department of Cardiology, Complejo Hospitalario Universitario de Pontevedra, 36701 Pontevedra, Spain; oscar.diaz.castro@sergas.es; 3 Research and Development, The Medical Center Foltra, 15886 Teo, Spain; pdevesap@foltra.org; 4 Scientific Direction, The Medical Center Foltra, 15886 Teo, Spain

**Keywords:** cardiovascular diseases, atherosclerosis, oxidative stress, angiogenesis and arteriogenesis, endothelial dysfunction, growth hormone, IGF-I, wound healing

## Abstract

This review describes the positive effects of growth hormone (GH) on the cardiovascular system. We analyze why the vascular endothelium is a real internal secretion gland, whose inflammation is the first step for developing atherosclerosis, as well as the mechanisms by which GH acts on vessels improving oxidative stress imbalance and endothelial dysfunction. We also report how GH acts on coronary arterial disease and heart failure, and on peripheral arterial disease, inducing a neovascularization process that finally increases flow in ischemic tissues. We include some preliminary data from a trial in which GH or placebo is given to elderly people suffering from critical limb ischemia, showing some of the benefits of the hormone on plasma markers of inflammation, and the safety of GH administration during short periods of time, even in diabetic patients. We also analyze how Klotho is strongly related to GH, inducing, after being released from the damaged vascular endothelium, the pituitary secretion of GH, most likely to repair the injury in the ischemic tissues. We also show how GH can help during wound healing by increasing the blood flow and some neurotrophic and growth factors. In summary, we postulate that short-term GH administration could be useful to treat cardiovascular diseases.

## 1. Introduction

The *hGH* gene family is composed by two growth hormone (GH) genes (*GH-N* and *GH-V*), and three placental genes that are located in the chromosome 17 [1]. It has been considered that the GH-V gene is expressed only in the placenta, although some studies indicated that this gene, or some other *GH* gene, still unknown, could also be expressed in the human pituitary gland [2,3]. In the case of GH-N, it is already well known that in addition to its pituitary expression, which is responsible for the actions of the hormone at the endocrine level, the hormone is also expressed in numerous cells and tissues, where it acts in an auto/paracrine manner [4]. Perhaps the heart is an exception to this peripheral expression of GH, as we will see later.

The regulation of GH pituitary expression is very complex, since in the last few years the classical knowledge of a positive regulation by GHRH and negative by somatostatin [5], has been changed after the knowledge of a series of factors that are decisively involved in that regulation [6]. This is the case, for instance, of the orexigenic Ghrelin, released by the empty stomach, or the postulated anti-senescence factor Klotho, mainly expressed in the kidney, but also in the brain and in the own somatotroph cells where it would act in an auto/paracrine manner for directly regulating GH secretion [7]; in addition, the growth differentiation factor 15 (GDF15), synthesized and released by cardiomyocyte, inhibits GH-induced hepatic expression of Insulin-like Growth Factor I (IGF-I), therefore inhibiting the IGF-I effect on hypothalamic somatostatin release and the direct negative effect of IGF-I on pituitary somatotrophs, thus acting as a coordinator between cardiac function and body growth or other IGF-I dependent GH effects on the human body [8].

**Figure 1:**
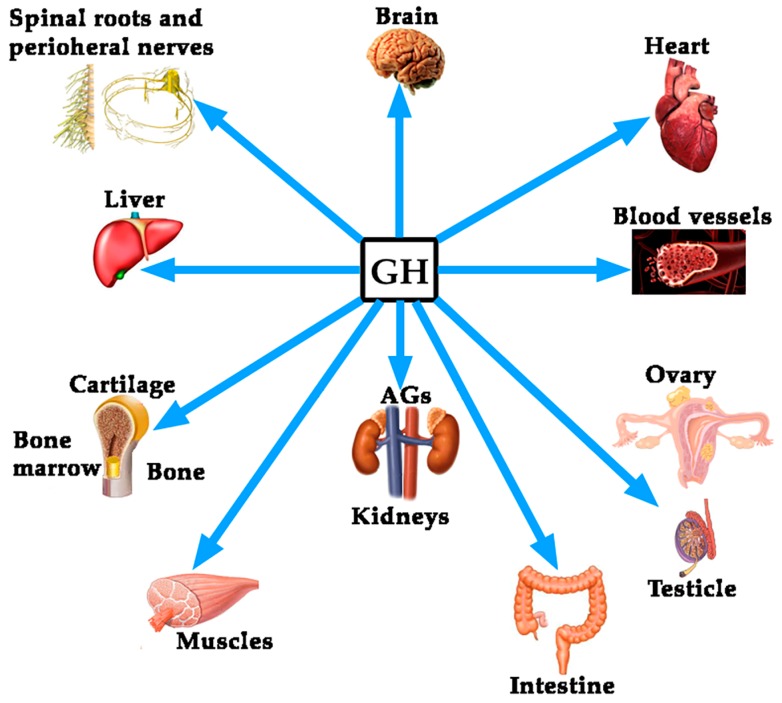
Growth hormone (GH) is a pleiotropic hormone acting on many tissues and organs in the human organism. Blue arrows show some of the most important territories in which the hormone produces positive effects. For a better understanding of this schema, see reference [4]. AGs: Adrenal glands.

Although the regulation of GH expression is not the aim of this review, perhaps the complexity of its regulation would explain the fact that far beyond of the concept that GH is mainly a metabolic hormone that is responsible for the longitudinal growth of the organism before puberty ends, the hormone exerts many other actions on practically all of the organs and tissues in the human body [4], as schematized in Figure 1.

In this review, we will focus on the effects of GH on the cardiovascular system; but before, it will be analyzed the role of the vascular endothelium as an internal secretion gland, as well as the main pathologies that affect the cardiovascular system, to subsequently assess the effect that GH can play in its treatment.

### 1.1. The Role of the Vascular Endothelium as an Internal Secretion Gland and the Effects of GH on It

Histologically, the vascular endothelium is a single unicellular layer that covers the internal surface of blood vessels and forms the wall of capillaries. However, despite its simplicity, this layer is very complex in physiological terms, since its location allows it to be able to detect alterations in the hemodynamic forces acting on the vascular wall (shear stress forces), as well as changes in circulating chemical signals, responding to all this by releasing vasoactive compounds, able to act oppositely depending on the signals received. For instance, at the level of hemostasis, the vascular endothelium can produce both anti-hemostatic factors (protein C, prostacyclin PGl2, tissue plasminogen activator, nitric oxide), or factors that favor hemostasis (von Willebrand factor, tissue Factor III, plasminogen activator inhibitor, thromboxane A2). The same occurs with the vascular tone, since vasodilators such as nitric oxide (NO) or prostacyclin (PGl2), and vasoconstrictors such as angiotensin II, endothelin (ET), thromboxane II and superoxide anion, can be released from the vascular endothelium to contribute to vascular homeostasis. Moreover, the vascular endothelium produces many growth factors, such as vascular endothelial growth factor (VEGF), platelet derived growth factor (PDGF), basic fibroblast growth factor (bFGF), and ET; but also inhibitory growth factors, as transforming growth factor-β (TGF-β). Even, the vascular endothelium can participate in immunological responses by producing interleukins (IL-1, IL-6 and IL-18), tumor necrosis factor-α (TNF-α), monocyte chemoattractant protein-1 (MCP-1), vascular cell adhesion molecule-1 (VCAM-1), intercellular adhesion molecule-1 (ICAM-1) and selectins E and P.

Most of these factors act locally by auto/paracrine mechanisms, so that they allow, as stated above, to regulate the vascular homeostasis.

In general, the vascular endothelium decreases the vascular tone, inhibits platelet adhesion and aggregation, decreases the activation of the coagulation system, stimulates fibrinolysis, decreases capillary permeability and inhibits the adhesion and migration of neutrophils and inflammation-generating macrophages. Therefore, endothelial dysfunction, a primary event in the development of atherosclerosis, is associated with increased smooth muscle vascular tone with arterial rigidity and elevated intima-media thickness.

Interestingly, while there are clear evidences that receptors for GH (GHR) and IGF-I (IGF-IR) are expressed in the vascular endothelium and myocardium [9,10,11,12], the possibility exists that GH itself is expressed in this territory, as in vitro studies reflect [13]. In any case, there are not doubts about the fact that GH plays a very important role on vascular endothelium. This statement is supported by early studies carried out in children with GH-deficiency (GHD), in whom GH replacement therapy recovered existing endothelial dysfunction. This is the case, for instance, of children with renal insufficiency; in them, endothelial dysfunction is quite common finding, but GH therapy reverses it [14]. In addition an improvement of the arterial response to induced vasodilation were observed in GH-deficiency (GHD) adolescents after GH-replacement therapy [15]; or in obese children, in whom obesity negatively affects the secretion of GH and constitutes a risk of developing atherosclerosis prematurely [16]. Similar results have been found in GHD adults (AGHD) after receiving replacement therapy [17], suggesting that GH reduces vascular inflammation, therefore reducing the vascular risk. Another study in AGHD patients showed that GH treatment led to a significant decrease in plasma levels of apolipoprotein B (Apo B) and C-reactive protein (CRP), while no changes were observed in IL-6 or on markers of endothelial function; but in all, GH administration decreased the cardiovascular risk in them [18]. AGHD patients show impaired coronary flow reserve, which is improved after receiving treatment with the hormone, suggesting that GH improves microvascular function and then could reduce cardiovascular morbidity and mortality in these AGHD [19,20]. A more recent study describes that six months of treatment with GH are enough to decrease cardiovascular risk and improve endothelial dysfunction [21].

One of the biomarkers for cardiovascular disease is the loss of circulating CD34+ cells [22]. CD 34+ cells are hematopoietic stem cells (HSCs) able to migrate to the bone marrow and give rise to all hematopoietic cell types when injected intravenously. They were first discovered in a cell surface glycoprotein [23], that is early expressed in hematopoietic and vascular-associated tissue. Therefore, CD34 is in fact a cell surface antigen able to act as an adhesion molecule but also as a facilitator of cell migration. These CD34+ cells are useful for treating many pathologies, including cardiac and vascular affectations [24], but also as a predictors of cardiovascular risk, since these cells collaborate in the maintenance of vascular homeostasis and repair, even in diabetic patients in which the circulating levels of CD34+ cells have been shown to be decreased [25]. CD34+ cells express receptors for GH and IGF-I, although the mechanism by which GH or IGF-I stimulates them has not been demonstrated, a parallel increase in granulocyte colony-stimulating factor (G-CSF) has been also observed [26]. Moreover, eNOS expressed by the bone marrow stromal cells influences recruitment of stem and progenitor cells [27]. In any case, the loss of circulating CD34+ cells observed in AGHD has been shown to be corrected after one year of GH treatment in AGHD, since the number of these cells increased and endothelial function improved [28]. This agrees with the fact that GH increases the production and release of endothelial progenitor cells (EPC) in non AGHD subjects, which in the vascular endothelium act as a repair cells [29]. Moreover, GH replacement therapy improves fibrinolysis in AGHD patients, most likely by increasing the release of endothelial tissue plasminogen activator as a response to venous occlusion [30]. On the contrary, other studies found that AGHD patients treated with GH showed increased concentrations of E-selectin, indicative of an uncorrected endothelial dysfunction [31]. These authors conclude that the beneficial effect of GH in these patients may be produced by the effects of the hormone on other mechanisms rather than acting on endothelial dysfunction.

As known, there are many ways to evaluate endothelial dysfunction, thus, influencing in the studies´ final results. For example, when diacron-reactive oxygen (DRO) metabolites or hemodynamic tests such as reactive hyperemia index are used, results are positive for the effect of GH on the correction of endothelial dysfunction in GHD [32]. In addition, it seems that both GH and E-selectin are not related, as, although the latter represents a type of cell adhesion molecules (CAM), and the hormone may increase them, GH rises VCAM but not E-selectin in GHD patients, being this an effect not dependent on GH-IGF-I.

The effects of GH administration on E-selectin had not been found in previous studies performed in healthy adults and AGHD patients [33]; instead vascular cell adhesion molecule-1 (VCAM-1) significantly increased in AGHD patients during GH treatment. Interestingly, serum from healthy patients treated with GH significantly increased the expression of VCAM-1 in cultured umbilical vein endothelial cells, suggesting that GH might act on VCAM-1 expression by an indirect mechanism, most likely related to the modulation of the expression of other circulating factors [33]. This might explain the reported negative effects of the hormone when administered to critically ill patients, since VCAM-1 mediates leukocytes extravasation which can lead to multiple organ failure in sepsis [34], although the increased mortality reported was observed with doses of GH quite higher (10–20 times) than usual treatment dose.

To our knowledge only one study reported no positive effects of GH replacement therapy on the endothelial dysfunction in AGHD patients [31], as only one report indicates that GH does not recover the endothelial impairment present in GHD children [35]. Perhaps, the small number of subjects, or the methodology used, or the time during which these studies were carried out justifies the contradictory results.

#### 1.1.1. GH, IGF-I, Klotho and the Vascular Endothelium

Klotho was first described in 1997 as a product of a gene that is involved in the suppression of several aging phenotypes in mouse. Initially, it was thought that Klotho would be implied in a signaling pathway regulating senescence and the severity of diseases related with the process of aging, such as atherosclerosis [36]. In mice, the gene codifies a membrane protein homologue to β-glucosidases, while in humans the gene has been shown to be composed of five exons and is located on chromosome 13q12. The gene suffers a physiological alternative RNA splicing giving origin to two transcripts, one of them being a membrane protein while the other one is secreted and predominates over the former [37]. The possible effects of Klotho on the physiology of the human vascular endothelium were first postulated in 1998, indicating that it protects the cardiovascular system by inducing NO endothelial production [38], although the possible mechanisms of action has not been clarified yet [39]. Further studies in mice of the same group demonstrated that secreted Klotho promoted endothelial increase of NO in aorta and arterioles [40], and that adenovirus-mediated *Klotho* gene delivery to a typical rat model of multiple atherogenic risk (OLETF rat) improved endothelial dysfunction, increased NO production, reduced increased blood pressure, and prevented medial hypertrophy, meaning that Klotho was a clear positive regulator of vascular function [41]. This was confirmed in *Klotho* mutant mice when observing that in these animals the density of blood capillaries was decreased at the tissue level and angiogenesis was impaired, as was the release of NO from the vascular endothelium [42]. These effects have been related to an action of Klotho on oxidative stress, responsible for inducing apoptosis and senescence in vascular cells [43]. While studies in animal models indicate a clear role for Klotho on the vascular endothelium, there are still no clear data on the physiological role that this hormone plays in humans on the cardiovascular system [44]. In vitro studies demonstrated that Klotho suppresses TNF-α-induced expression of intercellular adhesion molecule 1 (ICAM-1) and vascular cell adhesion molecule 1 (VCAM-1) in human umbilical vein endothelial cells, as well as the inhibition of eNOS phosphorylation induced by the administration of TNF-α [45], effects consistent with its previously postulated role in the modulation of endothelial inflammation. Klotho is mainly produced in kidneys; however, it seems that it could be expressed also in the vascular endothelium, with the only exception of endothelial cells from human brain [46]. In any case, Klotho is a circulating protein that increases NO production and protects the vascular endothelium [47,48].

**Figure 2:**
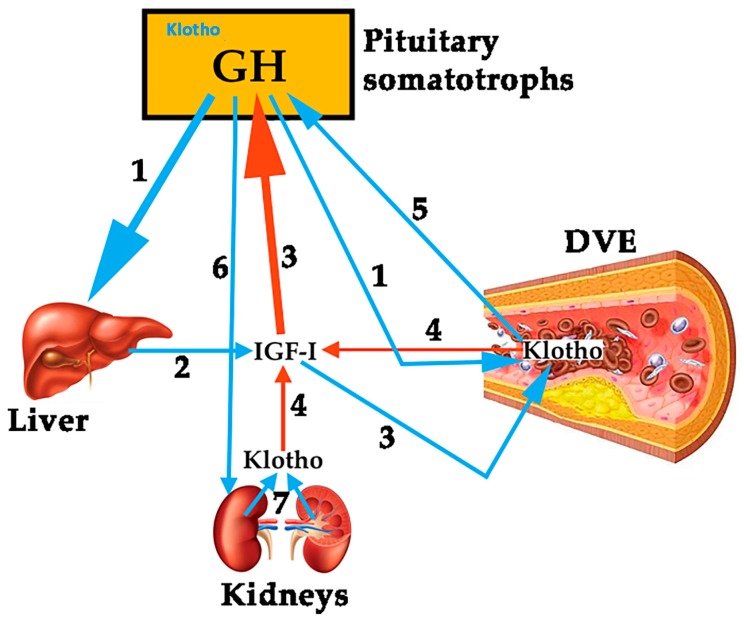
Schematic representation about the possible relationships between GH, Insulin growth factor (IGF-I) and Klotho, and its actions on the vascular endothelium. (**1**) Pituitary GH induces the hepatic expression of IGF-I (**2**) and acts on the repair of the damaged vascular endothelium (DVE), although it is also possible that the hormone enhances the production of Klotho by this damaged tissue. (**3**) Besides, its inhibitory effects on pituitary GH release, IGF-I also contributes to repair DVE, and, as in the case of GH, it could enhance Klotho production in DVE. (**4**) DVE secretes Klotho and it inhibits the negative effect of IGF-I on pituitary GH release, but plasma Klotho may also proceed from kidneys (**7**), contributing or being responsible for the inhibition of IGF-I effects on GH secretion. (**5**) The possibility exists that Klotho released from DVE stimulates GH secretion for repairing DVE. (**6**) GH plays an important role on the physiology of kidneys, being particularly important when there is a chronic kidney disease; since in this pathology there is a state of systemic Klotho deficiency, it is possible that GH tries to correct this problem associated to cardiovascular diseases. Some of these concepts are merely speculative, but existing data lead to think that there is a feedback regulation circuit between GH, IGF-I and Klotho. Blue arrows indicate stimulation and red arrows indicate inhibition.

To analyze the role of Klotho on the vascular endothelium is not the aim of this review. However, since it has been shown that this protein plays a role on pituitary GH secretion [7], we think it is important to try to establish a relationship between Klotho and GH, given that both are effective factors to prevent an injury to the vascular endothelium and repair it if a damage exits. Mice that do not express Klotho die early than normal mice showing many symptoms of aging, most of them typical of GHD [7]. Plasma levels of Klotho are low in GHD subjects, and the pituitary somatotrophs of Klotho-deficient mice are hypotrophic [7], suggesting that Klotho exerts a trophic effect on them. Besides this, Klotho-deficient mice are smaller than normal mice, and their GH-producing cells in the pituitary show lesser secretory granules [49]. In addition, Klotho strongly inhibits the negative effects of IGF-I on GH secretion, and increases GH secretion in cultured human GH-secreting adenomas [49]. All of these data indicate that Klotho is a positive active regulator of GH secretion, both in animal models and in humans. However, it is still unknown how GH and Klotho interact to repair a damaged vascular endothelium. For instance, in anorexia nervosa patients, in which the existence of an increased pulsatile secretion of GH is well known, while plasma levels of IGF-I are low or very low, due to malnutrition, plasma levels of Klotho are lower than expected for the age of the patients [50], but they increased significantly after the patients increased their body weight and, concomitantly IGF-I increased too. This suggests that IGF-I led to the increase of Klotho [50], perhaps for the maintenance of a physiological feedback loop between GH, IGF-I and Klotho. Those supposed relationships between the three hormones are schematized in Figure 2.

#### 1.1.2. GH, IGF-I and Ghrelin

The complexity of GH neuroregulation and the number of factors acting positively on the pituitary release of the hormone seems to be related to the multiple roles that GH plays in the human body, far beyond than those classically thought, such as the longitudinal growth of the organism before the puberty ends [4]. Interestingly, some of these roles are played in conjunction with the GH-stimulating factors. This is the case, for instance, of Ghrelin (GH-releasing peptide or GHRP), a small peptide found in the gastrointestinal tract in 1999 [51]. Curiously, besides its strong effects on GH secretion when administered intravenously, Ghrelin is an orexigenic hormone that is physiologically released when the stomach is empty. This is the reason by which patients with anorexia nervosa usually show increased plasmatic concentration of Ghrelin [52]. Therefore, it seems logical to assume that Ghrelin appears in evolution with a basic function: to induce eating behavior and optimize the use of immediate principles at the expense of promoting the release of an anabolic hormone, such as GH.

As described in the case of Klotho, Ghrelin also plays a hemodynamic role. In rats, there are receptors for Ghrelin in the aorta, left cardiac ventricle and left cardiac atrium. In healthy humans, the intravenous infusion of Ghrelin decreases the blood pressure, increases the cardiac index, and produces a greater volume of the pulse [53].

Interestingly, as it happens with GH, Ghrelin also plays a very important role in gastrointestinal processes that occur with inflammation, exhibiting gastroprotective properties. Perhaps in this area the most studied model is experimentally-induced pancreatitis in rats. For example, the administration of Ghrelin during the induction of acute pancreatitis in rats with normal secretion of GH, attenuated the development of pancreatitis by ischemia-reperfusion. On the contrary, in hypophysectomized rats, Ghrelin administration did not produce any beneficial effect, something that was achieved when IGF-I was administered. The authors of this study conclude that Ghrelin inhibits the development of ischemia-reperfusion-induced pancreatitis although this effect depends on the effects of Ghrelin on the secretion of GH and consequently IGF-I [54].

Similar conclusions were obtained after inducing acute pancreatitis in rats with cerulein, a decapeptide obtained from the skin of an australian amphibian, whose structure and actions are very similar to those of cholecystokinin. Cerulein induces an acute edematous pancreatitis, which in rats with normal GH secretion is reduced in its severity and producing a faster regeneration of the pancreas, being reduced the serum concentrations of the pro-inflammatory interleukin 1-β (IL-1β) and the serum activities of pancreatic enzymes amylase and lipase. In addition, pancreatic blood flow increased, as did pancreatic DNA synthesis. However, this did not occur in hypophysectomized rats, unless they received IGF-I in parallel to Ghrelin. This demonstrates that the protective effects of Ghrelin need of the existence of an adequate functioning of the GH-IGF-I axis [55]. Perhaps even more interesting is a recent study, in which an experimental colitis was induced in rats. Seven days after the induction of colitis (enema with acetic acid 3% in 1 mL) and treatment with Ghrelin, it was shown that the area of damage in colonic mucosa was clearly reduced in pituitary-intact rats, but increased in hypohysectomized animals. In addition, rats with normal GH-IGF-I production were shown to have enhanced blood flow in colonic mucosa while being treated with Ghrelin and increased mucosal cell proliferation, as well as reduced levels of IL1-1β and activity of mieloperoxidase. Just the opposite was found in hypohysectomized rats. Therefore, the authors concluded that while Ghrelin has a therapeutical effect in experimental colitis, this is mainly mediated by a normal activity of the GH-IGF-I axis [56].

In all, these concepts support our idea about GH as a hormone that plays many more roles in the body than those of a merely metabolic hormone and responsible for longitudinal growth until puberty ends.

### 1.2. Cardiovascular Disease as an Inflammatory Condition

Several diseases have been related to inflammation since many years, including atherosclerosis [57,58,59,60,61]. It is considered that inflammation plays a key role in atherogenesis, since it is not only involved in the development and progression of this process [61], but also in the associated symptoms [58]. Circulating monocytes and lymphocytes are present in the vascular wall early in atherogenesis, and both are responsible for the formation and complication of the atherosclerotic plaque [61].

A current study has demonstrated the high influence of inflammation in cardiovascular disease (CVD) from a clinical point of view. As is known, IL-6 has been previously associated with an increased risk of cardiovascular events, with independence of the cholesterol levels in plasma [57,62]. IL-6 amplifies the inflammatory cascade and is the main circulating cytokine linking systemic inflammation with local pathology [63,64]. It stimulates macrophages and promotes proliferation of smooth muscle cells (SMC) in atherosclerotic plaque [63], and stimulates coagulation by increasing messenger ribonucleic acid transcription of tissue factor and factor VIII [65]. IL-1β mediates the IL-6 signaling pathway [57], and canakinumab, a fully human monoclonal antibody targeting IL-1β, leads to a marked reduction of both, plasma levels of IL-6 and CRP, without lowering the level of low-density lipoprotein (LDL) in patients with diabetes who were at high vascular risk [57]. This drug led to a significant lower rate of recurrent cardiovascular events than the placebo [57].

The development of the atheromatous plaque is a multi-factorial process. SMC from the middle layer in the elastic arteries show a differentiated phenotype with a low proliferation and migration rate. Unlike the skeletal and cardiac myocyte, mature SMC may suffer a phenotypic modulation as a result of an atherogenic stimulus, with a re-entry in the cellular cycle. These activated states makes them proliferate and migrate to the vascular lumen, and synthesize some extracellular matrix (EM) components and proteases that modify the matrix, contributing to the atheromatous plaque.

The key aspect of the plaque formation is the endothelial dysfunction secondary to some atherogenic stimuli, such as hypercholesterolemia, hypertension, diabetes, tobacco, etc. As a consequence of this endothelial dysfunction, the inflammatory response is triggered. SMC are essential in the stability of these plaques. When there is a scarcity of them into the plaque, then the atheroma will be highly vulnerable to rupture. Plaque rupture and subsequent thrombus formation can lead to an acute event, although in the lower extremities this event can be better tolerated as a result of the numerous and large collateral network.

It is well known the role of LDL in this setting. Oxidized LDL (ox-LDL) have been related to the formation and complication of the atherosclerotic plaque [66]. LDL has high susceptibility of being oxidized. But, the oxidative environment in the vascular wall may also modify other lipids as HDL. Nicotinamide adenine dinucleotide phosphate (NADPH) oxidase, leukocyte- and platelet-derived oxidants, and red blood cell-derived iron-rich heme group, are part of the different systems implied in the oxidative modification of lipids, proteins, and DNA that in the vascular wall leads to atherosclerosis [66]. All of these oxidants maintain the inflammatory response and participate in the arterial wall rupture with platelet aggregation and thrombus formation.

Oxidative stress plays a main role in the origin of the pathogenesis of CVD. In a normal vascular wall, oxidative stress activates nuclear defense genes throughout the mediation of the nuclear factor erythroid 2-related factor 2 (Nrf2) [67]. This protects against the formation of foam cells by regulating the expression of antioxidant proteins and scavenger receptors [67]. Nevertheless, its function has not been properly understood, since a pro-atherogenic action has been also associated to Nrf2, because ApoE-null mice, which are deficient in Nrf2, develop smaller atherosclerotic plaques [68].

The recruitment of circulating leukocytes into the blood vessel wall is one of the major etiopathogenic mechanisms of atherosclerosis. This process is predominantly mediated by cellular CAM, which are expressed on the vascular endothelium and the leukocytes of the vascular wall, in response to atherogenic stimuli. In patients with peripheral arterial disease (PAD), increased levels of these integrins have been found during exercise, being associated with the severity and the extent of the arterial disease [69]. Antagonists of CAM have shown promise in treating inflammatory disorders in animal models [70,71]. Selectins, another group of integrins, are also elevated in PAD population. Studies with the anti-P-selectin antibody inclacumab in coronary arterial disease (CAD) have found a reduction in myocardial damage after percutaneous management [72]. This molecule also reduces elevated circulating platelet-leukocyte aggregates levels in PAD [73].

Exercise is associated with an increase in plasma levels of numerous inflammatory mediators in PAD, including thiobarbituric acid–reactive substances (formed as a byproduct of lipid peroxidation), thromboxane, IL-8, TNF-α, ICAM-1, VCAM-1, von Willebrand factor, E-selectin, and thrombomodulin [58]. Casual associations between biomarkers and PAD have not been established. However, inflammatory mediators can aggravate endothelial dysfunction, and markers, such as IL-6, are inversely correlated with maximum treadmill performance [74]. Although exercise acutely induces oxidative stress in patients with PAD, exercise training has consistently been shown to improve symptoms among these patients. In this sense, GH increases exercise performance, improving lean body mass, muscle mass, and cardiac output in AGHD patients [75]. Interestingly, exercise is a powerful inducer of pituitary GH release [5], most likely by inducing the hypothalamic delivery of noradrenaline that inhibits somatostatin, the main inhibitor of pituitary GH release [76]. As indicated, pituitary secretion of GH decreases while aging [5].

Endothelial dysfunction was recently associated with walking impairment independent of the ankle-brachial index (ABI), suggesting that endothelial dysfunction may contribute to the exercise impairment in PAD [77].

In addition, inflammatory mediators may also have proangiogenic and antiangiogenic effects, regulating the ischemic response [78]. In fact, patients with PAD have lower circulating VEGF-A and higher circulating inflammatory parameters of TNF-α and IL-8 when compared with controls with other comorbid conditions and cardiovascular risk factors [79].

**Figure 3:**
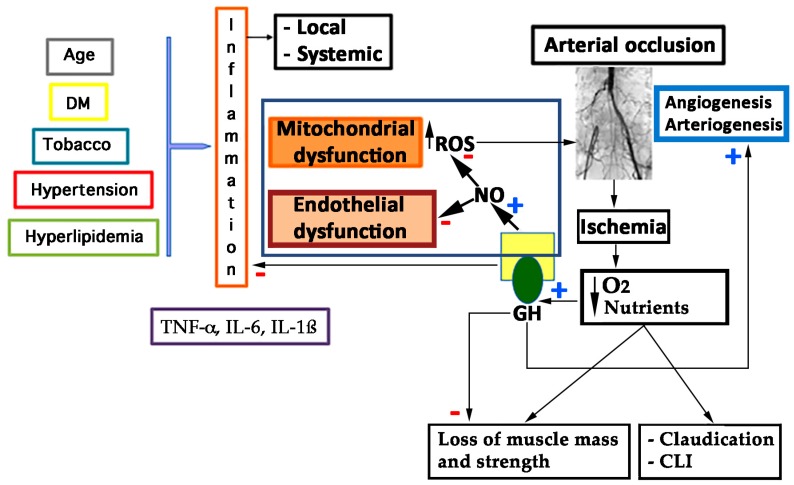
Cardiovascular risk factors converge to produce inflammation with increasing of TNF-α, IL-6, and IL-1β, which promotes endothelial and mitochondrial dysfunction, with the overload of ROS, all of them being responsible for atheroma plaque formation and arterial occlusion, which leads to hypoxia and decreased nutrition of tissue. Both factors contribute to the loss of muscle mass and strength and symptoms such as intermittent claudication or, critical limb ischemia. GH inhibits all of these deleterious effects from cardiovascular risk factors, promoting the NO pathway that compensates redox imbalance, corrects endothelial dysfunction (increasing endothelial-dependent vasodilation), decreases inflammation, and stimulates angiogenesis and arteriogenesis. NO: nitric oxide; ROS: reactive oxygen species; TNF-α: tumor necrosis factor alpha; IL: interleukin; GH: growth hormone; O_2_: oxygen; Blue crosses: stimulation; Red rectangles: inhibition.

On these bases, atherosclerosis is a complex process involving oxidative stress, endothelial dysfunction, inflammatory cell recruitment, platelet activation, and lipid deposition. Figure 3 schematizes these concepts.

Thus, to find biomarkers that can predict either the risk for suffering CVD or the risk for progression is of high interest, but this is not the aim of this review. Thereby, we will address those biomarkers related to GH that support its action and reduce the risk of CVD. Regarding the relationship between GH and vascular markers, most knowledge about this issue comes from the studies that were performed in acromegaly and GHD patients. As mentioned, significantly lower levels of VCAM-1 have been found in GHD patients than in healthy subjects; moreover, they increase during GH treatment, as compared with patients treated with placebo [33]. The development of GHD after the treatment of acromegaly affects adversely the body composition and inflammatory biomarkers of cardiovascular risk [80].

Visceral adiposity and lipids are one of the best studied markers in CVD. The increase of NO after GH administration lowers lipoxygenase activity and ox-LDL [4]. AGHD patients suffer an elevated risk of CVD because of hyperlipidemia, among other factors. GH therapy in these patients improves the lipid profile and decreases the vascular risk. The visceral fat is elevated in GHD children and adults, perhaps because GH produces lipolysis, and when GH is administered it reverts this increased adiposity [4,81]. Since GH secretion is deficient while aging, the progressive increase in both, fat stores and cardiovascular risk, seen in the elderly population could be due, at least in part, to the insufficient secretion of the hormone.

### 1.3. Coronary Arterial Disease (CAD) and Heart Failure

CAD is a broad term including several related syndromes caused by myocardial ischemia, an imbalance between cardiac blood supply perfusion and myocardial oxygen and nutritional requirements.

Cardiovascular disease (CVD) is the most important cause of death worldwide [82], and a major economic global burden [83]. Despite reductions in CVD mortality in high-income countries, global CVD mortality increased by 41% between 1990 and 2013, which was largely driven by rises in low-income and lower-middle-income countries [84]. Among CVD, coronary arterial disease (CAD) is the leading cause of death [82,83,84,85], and most of ischemic processes are usually of an atherosclerotic origin. Although there are other infrequent causes such as an anomalous origin of these arteries, its spontaneous dissection, or embolisms.

As indicated before, atherosclerosis implies a degenerative inflammatory process where different risk factors (diabetes, hypertension, dyslipidemia, smoking, obesity, sedentary lifestyle) damage the endothelium, favoring the entry of LDL particles that oxidize and initiate a complex inflammatory and fibrotic process within the arterial wall that culminates with the development of a plaque that can obstruct the coronary lumen, therefore preventing proper blood flow. Although the atherosclerotic process is usually chronic, abrupt plate instabilities can erode or ulcerate the endothelium giving rise to a thrombotic phenomenon that can obstruct the coronary artery suddenly causing an acute coronary syndrome (unstable angina or acute myocardial infarction). It is estimated that throughout the world these processes are responsible for approximately 7 million deaths per year, being the main cause of mortality in the population of industrialized countries.

The other major disease regarding the cardiovascular system is heart failure (HF); it affects about 2% of the adult population worldwide. Its prevalence is clearly age-dependent, ranging from less than 2% of people younger than 60 years to more than 10% of those older than 75 years, and it is estimated that it will increase by 25% in the next 20 years [86,87,88]. The etiology of HF is diverse and most patients have a history of hypertension, coronary artery disease, cardiomyopathies, or valve disease, or a combination of these [86,87]. HF has a poor prognosis, with high rates of hospital admission and mortality; costs related to the treatment of HF encompass 2–3% of the total expenditure of healthcare systems in high-income countries, and it is believed that they will increase by more than 200% in the next 20 years [88].

Ischemic cardiomyopathy remains the leading cause of left ventricular (LV) systolic dysfunction and HF. Some strategies to deal with this condition have been developed. Among them, treatment with GH, IGF-1, and natural and synthetic GHRP have been explored [89].

GH plays a key role for the development of a normal heart during fetal development, and plays a positive role in maintaining the structure and function of the normal adult heart, by stimulating cardiac growth and heart contractility [90,91,92]. The GH-IGF-I axis can modify cardiac activity and output, and regulate peripheral resistances [11]. The interactions between heart and GH are complex. In fact, it has recently been shown that the heart may influence body growth in pediatric heart disease. In these situations, cardiomyocyte synthesize and release GDF-15, which inhibits liver signaling by GH, therefore impeding the release of IGF-I and affecting body growth [8].

Life expectancy is reduced in patients with hypopituitarism as compared with healthy controls (two-fold higher risk of death for CVD, higher risk in women than in men). The main cause of death in GHD is HF [89], and the deficiency in GH has been considered one of the most relevant factors of the increased mortality in these patients [93,94].

Nowadays, despite multiple studies about the interaction of the GH-IGF-I axis and the cardiovascular system, the clinical importance of effects of GH and local and endocrine IGF-I in adults remains to be clarified.

### 1.4. Peripheral Arterial Disease

PAD is the term commonly used currently to refer to the atherosclerotic pathology affecting peripheral arteries of the lower extremity and compromising partially or totally the flow in them. Although less frequent that the other two main CVD, cardiac and cerebrovascular, it affects more than 200 million people worldwide [95]. Maybe, the spectrum of symptoms may vary from asymptomatic or atypical disease, to critical limb ischemia (CLI), the most severe form that threatens the limbs. However, the estimated prevalence depends on the tools used for the diagnosis. In people aged 60–70, the prevalence is about 8% in the Spanish population [96]. For those aged >70, it is generally accepted that the prevalence rises to 20%. Additionally, PAD is an independent predictor of cardiovascular mortality and morbidity [97].

#### 1.4.1. Endothelial and Mitochondrial Dysfunction in PAD: The Role of Oxidative Stress

As stated above, oxidative stress is the key aspect in producing the endothelial dysfunction that triggers the atherosclerotic process and the aging of the vascular system [66,98]. However, not only do vascular risk factors contribute to this phenomenon, but also the own exercise leads to the generation of superoxide-anion and other mediators of endothelial dysfunction, which has been correlated with the clinical severity of PAD [99]. This endothelial dysfunction is not only located in the major arteries, but also in the microcirculation of the skeletal muscle [58]. Patients with PAD suffer a constant ischemia-reperfusion syndrome as they walk and rest, generating reactive oxygen species (ROS) that affect muscle fibers [99], and impairs mitochondrial function, reducing the energy production [100,101]. In fact, higher carbonyl and 4-hydroxy-2-nonenal levels have been found in calf muscle samples, indicating the oxidative stress [102]. Skeletal muscle mitochondria releases free radicals during the ischemic process, including superoxide-anions and other ROS derived from the redox cascade [103,104]. Reperfusion also has the same effect, increasing the oxidative stress [104]. These ROS contribute to the endothelial dysfunction and the alteration of proteins in the skeletal muscle, and may lead to mitochondrial DNA injury in the long-term [105]. This DNA injury is also seen in less affected limbs of patients with unilateral PAD, suggesting that PAD is not only a local problem, but rather a systemic one [106].

Mitochondrial pathways are vulnerable to free-radical injury [107], and PAD patients show reduced activities of complexes I and III of the mitochondrial respiratory chain [108]. These observations suggest that electron transport chain activity is impaired in PAD, probably due to the ischemia-reperfusion injury and old age, which spreads the oxidative injury and the metabolic dysfunction. Lactate levels are also significantly elevated in PAD skeletal muscle, because of an anaerobic oxidation of glucose, a decreased pyruvate dehydrogenase activity [109]. At this point, it is of interest to remark that GH is a mitochondrial protector [110,111,112], therefore suggesting that the hormone may play a positive role in this process, since GH restores the redox imbalance, improving mitochondrial respiratory chain and the production of energy.

Endothelial dysfunction has been evaluated in Japanese patients with AGHD in the GREAT study. After 24 weeks of GH replacement therapy, the hormone significantly lowered plasma diacron-reactive oxygen metabolites and improved endothelial function, as measured by reactive hyperemia index [32]. This indicates that GH can exert a protective role in redox balance in AGHD, in which predominates a pro-oxidant environment increasing the atherogenic risk, but this is corrected by short-term GH administration without fully normalizing IGF-I levels [113]. Moreover, GH has a role in stress resistance by altering the functional capacity of the glutathione S-transferase (GST) system through the regulation of specific GST family members in long-living Ames dwarf mice [114]. The hormone also affects the regulation of thioredoxins (TRX) and glutaredoxins (GRX), which are factors that regulate post translational modification of proteins and redox balance, thereby further influencing stress resistance [114]. However, the exact role of GH in redox balance has not been completely understood, as in oxidative stress-induced conditions may enhance oxidation [115]. Thereby, both GH overproduction and deficiency are tightly linked with enhanced oxidative stress.

#### 1.4.2. Endothelin (ET) and PAD

Endothelial dysfunction might be also traduced by an imbalance between the endothelium-dependent vasodilation, as mediated mainly by NO, and vasoconstriction, as mediated mainly by ET.

It has been well documented that vascular ET production is elevated in atherosclerosis and influences the development of atherosclerotic lesions through a variety of mechanisms [116]. ET participates in several key steps in the inflammatory component of atherosclerosis, increasing various cytokines from monocytes [117], and enhancing the uptake of LDL by these cells, thus promoting foam cells [118].

GH has been broadly related to an increase in the production of NO, improving vasodilation. But, GH is also related to ET, as an increased secretion of GH and Ghrelin have been demonstrated in cattle after the injection of ET-1 and ET-3 [119,120]. Therefore, GH increases physiologically in response to the increased level of ET. Despite that the relationship between GH and ET has not been well established yet in CVD, it seems that GH may compensate the deleterious effects of ET, as the treatment with the hormone improves ET-induced stroke in adult rats [121]. Perhaps this is due to the actions of GH on NO production.

## 2. Discussion

As described, GH plays a key role for the development of a normal heart during fetal development, and plays a positive role in maintaining the structure and function of the normal adult heart, by stimulating cardiac growth and heart contractility, but also the structure of a normal vascular endothelium. Therefore, a point of interest to analyze how GH may affect the cardiovascular system is the study of its effects on the vascular endothelium.

### 2.1. The Role of GH in the Vascular Endothelium

Two conditions in which the effects of GH on endothelial dysfunction may provide interesting data are acromegaly and aging.

In the case of acromegaly, plasma levels of two biomarkers of endothelial dysfunction and atherosclerosis, such as endothelin-1 (ET-1) and total homocysteine levels (tHcy), were measured in patients with active acromegaly and cured disease [122]. While tHcy was similar in both groups of patients, ET-1 was significantly higher in active acromegaly, suggesting that it contributes to premature atherosclerosis and cardiovascular affectations that were observed in this pathology, although the role that is played by IGF-I on these vascular affectations could not be discarded. On the other hand, patients with acromegaly, despite presenting a higher incidence of other cardiovascular risk factors (hypertension, insulin resistance), do not present a clear excess of CAD or stroke in comparison to normal counterparts [4,11,123].

Particularly important, in our opinion, is the case of aging. Important changes in pituitary GH secretion along the life have been widely described (for a more detailed comprehension, see references [4,6]. An exponential decline in plasma GH concentrations starts from 18 to 30 years of age, until it is practically imperceptible in elderly subjects, a phenomenon known as somatopause. In this situation, plasma levels of IGF-I are also low, although the liver production of this peptide depends not only on GH but also on the nutritional status of the organism [4]. Aging is associated with an increased risk of atherosclerosis, but we know now that this disease can begin earlier, during youthfulness. It has been proposed that the increased risk of atherosclerosis as we age, is due to low production of EPC, which makes unable to repair atherosclerotic vascular walls [124]. Treatment with GH during 10 days led, in middle-aged subjects to an increase in plasma levels of EPC which, moreover, improved in its capacity to migrate and incorporate into tube-like structures, and showed an increased endothelial NO synthase (eNOS) expression up to levels equivalent to those of healthy young subjects. That is, GH treatment decreased EPC senescence and increased telomerase activity. In the same study, aged mice treated during seven days with GH or IGF-I increased EPC levels and ameliorated EPC functions. This was not observed when GH treatment was given during only two days. Results from that study attributed to IGF-I, rather than to GH, the reversal of age-dependent EPC dysfunction [124]. We do not know whether these results appear as an IGF-I age-related effect, but other studies, as described before in healthy young people, indicated that GH effects on the vascular system are not dependent on IGF-I, postulating that GH acts directly on GHR and eNOS in the vascular endothelium [9]. These contradictory results led to us suggesting that GH administration during somatopause does not produce clearly favorable effects on the endothelial dysfunction, while combined treatments with GH plus IGF-I may produce more beneficial effects on the vascular wall in elderly individuals [125]; however, we do not think that this combination is advisable.

Preclinical studies in hypophysectomized rats also showed that the lack of GH production is associated with the development of atherosclerosis [126], while GH treatment during two weeks reversed several biomarkers indicative of the developing arterial disease. These researchers identified in the aorta of hypophysectomized rats 18 genes that were regulated by GH, which most likely have a physiological effect on vascular tone and atherogenesis. Among these genes, they found that GH induced an increased expression of the KATP channel, which plays a key role in the regulation of vascular tone, therefore involving GH in this regulation [126]. However, plasma levels of GH must be within normal ranges, since, as it occurs in acromegaly, transgenic mice overexpressing bovine GH develop an endothelial dysfunction, which depends on the age of the animal and the type of blood vessel, indicating that the affectation in endothelial function is most likely produced by increased production of mitochondrial ROS, followed by many other affectations in vascular function [127].

Curiously, similar results to these shown in transgenic mice overexpressing GH, have been reported in hypopituitary Ames dwarf mice aortas in terms of enhanced production of ROS and lesser expression of antioxidant enzymes (for instance, glutathione peroxidase and eNOS), therefore leading to vascular oxidative stress [128], a first step, as stated above, to develop endothelial dysfunction. Similarly, peripubertal GHD in Lewis dwarf rats leads to a pro-oxidative cellular condition most likely responsible for the development of an altered vascular phenotype (in both structural and functional terms), which leads to vascular affectations, early accelerated, later in the life of these animals [129]. GH treatment reverses these impairments that, interestingly, do not occur equally in the cerebral vessels than in the aorta of these genetically dwarf rats [129]. Another model for analyzing the effects of GH on the vascular system comes from studies in which rats are undernourished during pregnancy. Maternal undernutrition produces increased blood pressure and endothelial dysfunction in adult offspring, but if pups receive early pre-weaning GH treatment (from day 3 after birth until weaning in day 21), then adult vascular function is normal; this contrast with what happens in the offspring that received saline during these days before weaning. This indicates that early GH treatment can reverse the vascular alterations resulting from maternal undernutrition during pregnancy, but also that there is a developmental cardiovascular programming, which is susceptible to be reversed by early treatment with GH after delivery [130].

While results from both preclinical and clinical studies clearly indicate that GH plays a key role in the prevention or recovery of endothelial dysfunction, it is not clear at all which of the effects of GH are due to a direct action of the hormone and which are mediated by IGF-I, because this peptide and its receptors (IGF-IR) are widely expressed in endothelial cells [131]. Moreover, GH induces the expression of IGF-I in many territories, including the fetal brain [132]; however, GH seems to be unable to increase the transcription of IGF-I in endothelial cells, and, in fact, systemic or local infusions of GH lead to a prompt increase in forearm blood flow and NO release in healthy humans, without increasing plasma IGF-I concentrations or muscle IGF-I expression [9,133]. The fact that it seems that GH is produced by endothelial cells, and endothelium-derived GH stimulates the proliferation, migration, survival, and capillary formation of endothelial cells in an autocrine manner [13], clearly indicates that the hormone exerts direct effects on the vascular endothelium, although IGF-I is also vasoactive activating eNOS via Pi3K/Akt [134], a signaling pathway also used by GH, as we first demonstrated [135] and further checked when analyzing GH signaling pathways in neural stem cells from nine-days old mice [136]. More recently, the knowledge about GH signaling has been significantly improved by the group of Carter-Su [137].

Perhaps some of the apparently contradictory results here reported, in relation to the lack of effects of the administration of GH on the vascular endothelium, in AGHD and GHD children [31,35], and the attribution to IGF-I rather than to GH the positive effects on the vascular wall, proceed from the recently described relationships between GH, IGF-I, and Klotho [7,49], but also on the effects of Klotho on the vascular endothelium and aging.

The impact of GH on inflammatory processes is not well understood yet. Evidence shows controversial data of both anti- and pro-inflammatory effects of the hormone. GH therapy reduces the levels of CRP in GHD patients [138], and exerts anti-inflammatory effects in different experimental models of sepsis by lowering TNF-α [139]. Exogenous GH also may improve the effects of sepsis-induced IGF-I resistance [140]. Conversely, a massive increase of GH in GH transgenic mice has a pro-inflammatory effect, thus increasing pro-inflammatory cytokines [140]. Pro-inflammatory effect of GH seems to be secondary to its indirect action on CAM, mediated by VEGF among other factors. VEGF has been described as a strong-inducing agent of CAM on endothelial cells during inflammation [141]. Given the fact that GH directly increases VEGF levels after its administration, this peptide could be one of the main mediators of the effects of GH. It seems, thereby, that high supraphysiological administration of exogenous GH could increase inflammation, while doses that are used for treating GHD, or even short-term GH administration to non-GHD patients, may represent a protective factor against this issue.

The role of CRP, IL-6, and TNF-α in CVD has also been well established [59,64,142]. In AGHD patients, the administration of GH decreased CRP and IL-6 levels, some which did not occurr when treating them with placebo [143]. However, another study in AGHD, showed that GH therapy also reduced CRP, but failed in reducing TNF-α and IL-6 levels [138].

Pregnancy associated plasma protein A (PAPP-A) has been recently included among markers of cardiovascular risk being associated both to the presence of carotid atherosclerosis and acute coronary syndrome [144,145]. PAPP-A is also significantly elevated in AGHD [146], and GH replacement therapy decreases this specific and not generic biomarker of CVD, although not in all GHD patients [147].

At this point, the own GH could be a marker, as the deficit of both GH and IGF-I leads a more aggressive heart failure, with impaired functional capacity and poor outcomes [148].

All of these data support the possible role played by GH in the correction of the state of inflammation in patients suffering from CVD, at least in those with AGHD. The protective effect of GH in inflammation is secondary to its action against oxidative stress, most likely as a consequence of its action on NO and extracellular signal-regulated Kinase (ERK) pathway.

**Figure 4:**
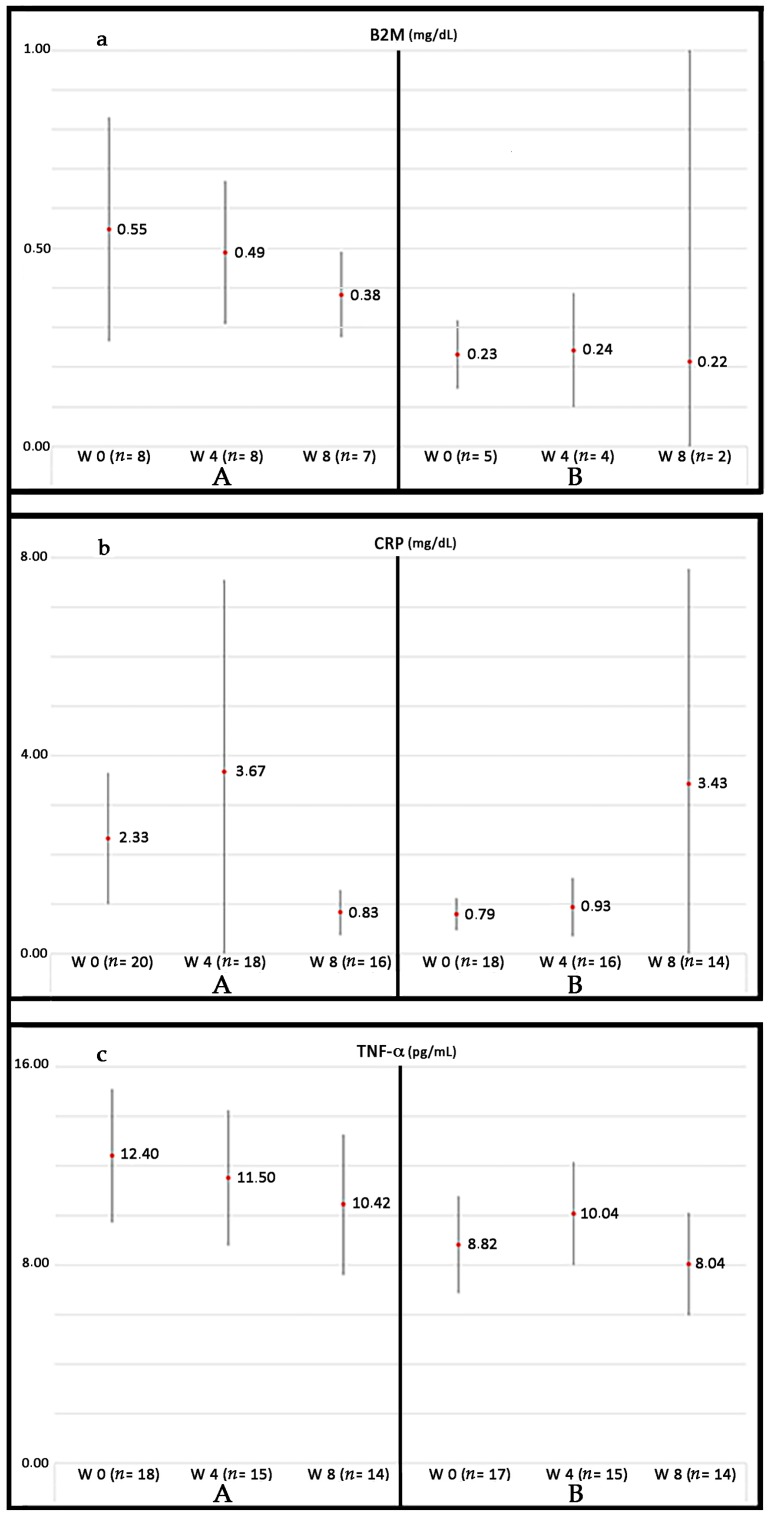
Evolution of some biomarkers of inflammation analyzed in the GHAS study. Values are shown as the mean ± SD. (**a**) evolution of plasma levels of B2M (β-2-microglobulin) throughout the treatment with GH (A) or placebo (B); (**b**) evolution of plasma levels of CRP (C Reactive Protein) and (**c**) TNF-α, throughout the two groups of treatment. (A) and (B) represent different groups of treatment (GH or placebo, respectively). Note the tendency to decrease in group (A) as compared with the group (B). Patients from group (A) had significant higher basal levels of markers, indicating that patients in this group suffered from a more severe inflammatory disease as compared with group (B). Significant differences in the end of the study have not reached because of the small sample of patients still analyzed (note the differences in *n*). W = weeks of treatment. *n* = number of patients analyzed until now.

Currently, our group is conducting a phase III randomized controlled trial (RCT) in patients suffering from PAD without options for revascularization: Growth Hormone Angiogenic Study (GHAS), Eudract 2012-002228-34, approved by the Spanish Agency of Drugs and Health Products (AEMPs), and the Autonomic Committee on Research Ethics in Galicia (CAEIG, 2012/378), Spain, in which patients receive GH or placebo. Although this study is already finished and results from it only began to be evaluated, early results show that within the markers used, TNF-α is the most frequently elevated in these patients (74%), followed by β2-microglobulin (69%) and CRP (60%). Figure 4 depicts the graphic tendencies that show these patients depending on the group of treatment.

### 2.2. GH and Coronary Arterial Disease

The effects of GH-IGF-I in the incidence and prognosis of CAD are controversial.

**Figure 5:**
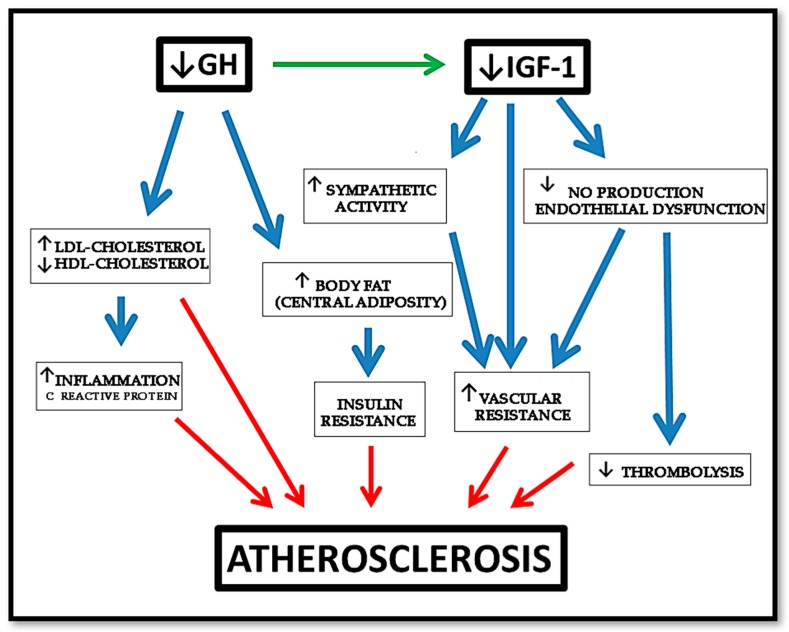
Effects of GH deficiency on atherosclerosis. GH: Growth Hormone, IGF-1: Insulin growth factor 1. NO: nitric oxide. Blue arrows indicate the effects produced by decreased GH secretion, while red arrows indicate how atherosclerosis is developed. Black arrows indicate increase or decrease, depending on the address of the arrow. Green arrow indicate that the lack of GH leads to decreased IGF-I secretion.

As described before, GHD is associated with an increased prevalence of atherosclerosis, CAD, and stroke caused by an increased prevalence of atherosclerotic risk factors, such as alterations of body composition, lipid profile, and coagulation pattern [4,11,123], as shown in Figure 5.

The changes in lipid profile observed in AGHD consist of increased in LDL and triglycerides, and decreased in high-density lipoprotein (HDL) (the latter observed only in women), with no differences in lipoprotein (a) [149]. GH replacement positively reverses this negative lipid profile. In addition, a decrease in CRP has been observed in these patients after GH therapy, while no clear changes seem to be produced in circulating triglycerides [149,150,151]. However, no study has determined whether GH has an additive effect that optimizes statin therapy; therefore, this remains an open question.

Regarding hypertension and peripheral resistance conflicting results have been reported in the literature [123]. Hypertension is quite frequent in GHD patients, and this condition results in impaired vasodilation responses to stress and/or exercise. As described, the GH–IGF-I axis reduces vascular tone by several mechanisms [152], although some vasoactive effects of GH may also have a central origin. In fact, GHD patients have markedly increased muscle sympathetic nerve activity, and GH replacement therapy has been shown to reduce it, suggesting an involvement of the GH-IGF-I axis in the autonomous sympathetic system regulation [153].

In some AGHD (patients with high base-line diastolic blood pressure, such as elderly GHD patients or those with previous Cushing disease), GH replacement reduces blood pressure, whereas in other patients (especially in young GHD patients), no changes in blood pressure have been shown [123,154].

Besides the cardiovascular risk factors mentioned above, GHD patients were shown to have increased blood vessel intima-media thickness (IMT) that is well known to represent one of the earliest morphological changes in the arterial wall in the process of atherogenesis [155]. It has to be highlight that femoral and carotid arteries IMT are independent predictors of CAD extent [156].

A decrease in IMT has been shown in several studies after the administration of GH to GHD patients [157]. Increases in IMT predict the development of symptomatic coronary disease, thus GH treatment may have a significant improvement in cardiovascular outcome, but this question has not been specifically analyzed in patients with GHD.

Regarding hard clinical endpoints, we previously commented on the increased risk of cardiovascular mortality in GHD patients. The worse cardiac risk profile (mainly hyperlipidemia) of these patients may explain part of the excess in CAD and mortality, but the studies do not allow for obtaining a definitive conclusion. As stated, GHD patients also has an increase in visceral fat, which decreases in response to GH therapy within six months after the initiation of the same, and it is maintained if the treatment is continued.

Despite all of these facts, there are no prospective, long-term randomized studies in AGHD patients comparing GH treatment to placebo on cardiovascular hard outcomes and mortality, and it is likely that there will never be such a study. A more recent and prospective trial found a lower mortality in GH treated hypopituitary patients when compared with a retrospective analysis of patients who had not been treated with GH [158]. However, again, the different time periods covered also included dramatic changes in the treatment of risk factors such as hypertension, diabetes mellitus, and hypercholesterolemia.

Some experimental models of CAD have shown the possible benefit of the GH-IGF-I axis on angiogenesis. For example, in rats with myocardial infarction (MI), inducing myocardial overexpression of IGF-I by delivering a human *IGF-I* gene by means of an adeno-associated viral vector led to angiogenesis [159]. This study demonstrated that the angiogenic process, measured by micro-SPECT-CT 16 weeks after administering the gene, persisted over time, leading to an improvement of the capillary network in rat hearts, a decreased left ventricle remodeling and an improved cardiac function. In rats with large MI, the early application of recombinant human GH (rhGH), starting three days after MI, attenuated LV remodeling without LV hypertrophy [160]. When administered a combination of GH and IGF-I, beneficial effects of early treatment have also been reported on infarct size, survival and cardiac gene expression after acute MI [161]. Curiously, these benefits were no observed when, instead of rhGH, recombinant rat GH (rrGH) was utilized [162]. This seems to be contradictory, as rhGH in rats might produce anti-GH antibodies and fail. Heterogeneous observations in experimental models can be explained by several facts, such as the early or late GH treatment, the different origin of HF, the different dosing regimens and short duration of treatment [89].

### 2.3. GH and Heart Failure

As stated, GH plays an important role during myocardial development that can easily be seen in untreated GHD children. They present cardiac atrophy with a reduction in the LV mass, ejection fraction, and cavity dimensions, as well as reduced cardiac output, high peripheral vascular resistance, and reduced functional capacity when compared with healthy controls of the same age, sex, and height [163]. When GHD appears in adults, it does not produce a reduction in cardiac mass, but cardiac performance and exercise capacity are impaired [164].

On the other hand, GH excess exerts different and opposite effects on the heart. In early-stage, it enhances cardiac performance, whereas it causes fibrosis and cardiac dysfunction in the intermediate-late phase. This apparent discrepancy is easily clarified: a physiological GH level or short-term excess exert positive inotropic effect; whereas long-term exposure to GH excess induces cardiac dysfunction and progression to heart failure by causing morphological and functional adaptive changes [165]. The most relevant histological abnormalities are interstitial fibrosis, reduced capillary density, increased extracellular collagen deposition, myofibril disorder, lympho-mononuclear infiltration, and myocyte death due to necrosis and apoptosis [165,166].

GH may regulate cardiac growth and metabolism by increasing protein synthesis (troponin I, myosin light chain-2, and actin), and cardiomyocyte size, increasing collagen synthesis and promoting cardiac hypertrophy [166,167,168]. IGF-I may reduce apoptosis of cardiomyocyte, thus preventing myocyte loss [167]. The GH-IGF-I axis can also increase cardiac contractility by enhancing calcium sensitivity and reducing vascular resistance [164,166].

Chronic heart failure (CHF) patients have a prevalence of 30% in GHD, and this fact identifies a subgroup of CHF patients that are characterized by impaired functional capacity, left ventricle remodeling, and elevated natriuretic peptide levels and increased all-cause mortality.

Several groups have studied the effects of GH and IGF-I in patients with HF of different origin, mainly CAD. GH replacement trials show an increase in left ventricle (LV) mass and improvement in cardiac performance, diastolic filling, and systolic function after GH treatment in children or adults with GHD. Nevertheless, randomized placebo controlled studies show conflicting results, with an increase in LV mass related to serum IGF-I levels, but no change in LV wall stress, arterial blood pressure, ejection fraction, clinical status, or 6-min walking distance. While different results with different regimes of GH treatment have been reported in HF patients, RCT did no observe any benefit at the dose of 0.17–0.67 mg/day for 26 weeks of rhGH [89]. However, Napoli et al. observed improvement of the endothelial function in 16 patients treated with 1.33 mg of GH every second day after a period of three months [169], highlighting the potential role of GH in delaying the progression of HF. GHRP studies also reached a slight profit in these patients [53]. The conflicting results of the clinical trials with GH in HF may be related to the small number of patients enrolled, the different dose and duration of GH treatment, the different cardiac heart failure etiologies, and differences in the patients’ clinical characteristics. Besides, the discrepancies may also reflect the heterogeneity of IGF-I increase in response to GH treatment. In fact, a recent meta-analysis confirms that there is a clear relationship between changes in IGF-I concentrations achieved and the beneficial effects of GH treatment. Only in the trials in which IGF-I increased >89% from baseline levels, there were a significant improvement in cardiac performance, echocardiographic parameters and exercise capacity; whereas in the trials in which the increase in IGF-I was <89%, beneficial cardiovascular effects were not observed [170].

Given its possible positive effects on heart in “responders” patients, it could be speculated that GH treatment may be useful in some patients with HF, but more investigation is needed in this field.

### 2.4. GH and Molecular Aspects of Cardiovascular Risk Factors in PAD

Despite the negative epidemiological impact of cardiovascular risk factors, its main mechanism of damage is not completely clarified. However, it seems that they may modify redox balance. Since the specific role of each cardiovascular risk factor in redox balance has been widely described, as well as, the benefit of their treatment, we will only underline the main aspects related to GH and its possible role in diabetic patients.

As described before, GH therapy improves arterial hypertension in GHD patients by acting on the vascular smooth muscle ATP-sensitive potassium (KATP) channel, and on the lipid profile with independence of IGF-I.

A dysfunction of eNOS in both endothelial cells and platelets has been found in diabetic patients, which attenuates arterial remodeling [171,172]. But, the latter is also affected because of the lower sensitivity for the shear stress signals that these patients show. This aspect seems to be secondary to the massive calcification and multilevel arterial disease, as well as to the elevated vasomotor tone found that impairs the response to the vasodilator stimuli, and the enlargement of collateral arteries [171,172,173]. Moreover, a quantitative and qualitative alteration in EPC has been described in DM [171,174]. All of these factors explain both the strong atherosclerotic injury and the low capacity of compensating the latter after an arterial occlusion.

In addition, a high rate of patients with DM may suffer neuropathy. In these patients, the level of expression of several growth factors, such as neurotrophic factors, insulin-like growth factors, cytokine-like growth factors, and VEGF, are altered [174]. Secondary to neuropathy, the sympathetic nerve activity is usually altered.

GH could aid in the recovery of some of these deleterious aspects in diabetic patients, since, once again, the hormone increases eNOS production, decreases vasomotor tone, and may improve the nerve injury, by increasing neurotrophic factors, such as brain derived neurotrophic factor (BDNF). Moreover, GH can increase Substance P (SP), which is one of the main molecules implied in nerve damage and wound healing. SP and GH are strongly related [175,176]. In fact, GH improves wound healing in diabetic rats and mice [177], and SP could be one of the possible mediators. Since a high rate of diabetic patients have small vessels disease, angiogenic therapy with growth factors might be a good option for them.

Although GH may cause hyperglycemia or abnormal glucose tolerance, this is not a contraindication for using the hormone in diabetic patients, since clear benefit of GH therapy has been described in these patients.

### 2.5. GH, Age, and Cardiovascular Disease

The relation of GH and age requires a specific section, since age associates with both atherosclerosis and GH deficiency.

Aging is a biological process that causes the progressive deterioration of structure and function at the cellular level over time. Both cardiovascular structure and function are under a continuous remodeling process as we age.

There are two aspects responsible for the pathophysiological changes in the aged vascular bed: the impaired intrinsic cellular mechanisms to resist ischemic injury [178], and the impairment of vascular angiogenic capacity and endothelial function [179]. Evidence from animal model of hind limb ischemia has demonstrated a reduced capability to protect tissues from ischemic insult, as well as an impaired ability to establish collateral circulation in the aged animals when a major artery is occluded [180]. Aging may attenuate both angiogenesis and arteriogenesis, producing less proangiogenic cytokines or increasing the expression of antiangiogenic factors [180]. For instance, TNF-α, which promotes apoptosis in endothelial cells, is upregulated in cultured aged endothelial cells [181], and thrombospondin (TSP), a substance with antiangiogenic effect, increases its expression in healthy aged tissues [182].

EM plays a key role in angiogenesis, and aging alters the expression of metaloproteinases, integrins and structural proteins in EM. Tissue inhibitor of metaloproteinases (TIMP-1 or TIMP-2) has a negative impact on angiogenesis process. Higher levels of TIMP-2 were found in the aged endothelial cell lines than in the young ones [180].

During angiogenesis, EPC also play an important role. Both mice and human subjects have an age-dependent impairment of EPC. Middle-aged and elderly subjects had lower circulating CD133+/VEGFR-2+ EPC, with impaired function and increased senescence [124].

Arteriogenesis is also affected with aging, as collateral blood flow expansion has been seen to be delayed in aged rats with bilateral femoral artery occlusion [183], perhaps because of an attenuated sensitivity of the receptors for shear stress secondary to a less activation of Rho pathway, one of the main arteriogenic signal pathways [180].

Vascular aging is also characterized by increased mitochondrial ROS production in endothelial cells, which, in turn, decreases the bioavailability of the vasodilator and anti-apoptotic NO, increases cardiac oxygen demand, and promotes vascular inflammation by inducing nuclear factor κ-B (NF-κB). Both the expression and the activity of eNOS has been shown to be decreased in the cardiovascular system of aged animals [184] and in cultured aged human umbilical vein endothelial cells [181].

At the skin level, aging alters tissue inflammatory response slowing down wound healing [185].

In spite of these deleterious consequences of aging, elderly people still respond to physical or biochemical stimuli (exercise or exogenous angiogenic growth factors), which improve the angiogenic and arteriogenic responses. NO-donors as nitrates, and angiogenic growth factors, work equally in the ischemic tissues of animals of old age [180].

Aging might be a factor that contributes to the unsatisfactory results (not better than the placebo group) of recent clinical trials that are intended to expand collateral vasculature in the ischemic legs with angiogenic growth factors [186].

It has been highlighted before that aging also has a strong impact on GH production [187]. This fact can contribute to an imbalance between pro- and anti-angiogenic factors, favoring the latter. GH administration to old animals and humans raises plasma IGF-I, increases skeletal muscle, and improves immune and cardiovascular functions. Therefore, the relationship between age-related changes in cardiovascular function and the decline in GH levels with age is awakening interest. Both, in aged mice and in humans, the hormone reverses many of the deficits in cardiovascular function [187].

All of these are some of the reasons why the administration of GH to elderly people has been proposed [188], though it must be balanced with its possible side effects.

A possible good strategy to prevent cardiac aging, comes from the fact that both GH and Melatonin have antioxidant properties, therefore decreasing oxidative stress and apoptosis [189]. In fact, in this study in senescence-accelerated mice, it was proven that the administration of GH and Melatonin led to a clear reduction in the age-related changes in senescence-accelerated prone hearts. Interestingly, the additive effects obtained when these hormones were given together, were different to those induced when each hormone was given alone. These authors conclude that GH and Melatonin may be useful agents for counteracting oxidative stress, apoptosis, and inflammation in the aging heart. We fully agree with this proposal, mainly when we currently know the large number of beneficial effects that melatonin plays in the body (cardioprotective, neuroprotective, chelating of highly toxic free radicals, etc.), although at doses that are much higher than those used for jet lag. Moreover, GH is a mitochondrial protector.

### 2.6. GH and Neovascularization: Experimental and Clinical Evidences

That GH has a positive role on the vascular system has been broadly set out here and demonstrated in experimental and clinical studies. One typical example is that cerebral microvasculature decline with age is parallel to that in GH-IGF-I, and the administration of the hormone to aging rats increases the number of cerebral cortical arterioles [190]; or, as mentioned above, the novel role discovered for the hormone in stimulating wound healing, mainly as a consequence of its angiogenic action and its capacity for promoting myofibroblast differentiation [177,191]. In fact, the skin of GHD patients has reduced capillary density and permeability, which improves after they receive GH treatment [192].

One of the more attractive action of GH related to this issue is the mobilization of EPC into the bloodstream [29,124]. It seems that this action is not a direct stimulation of bone marrow, but an indirect effect via VEGF, SDF-1, or erythropoietin (EPO), among others [29]. It has been demonstrated an impairment of EPC with aging, and that the latter is corrected after GH therapy mediated by IGF-I.

GH may have direct, non-IGF-I mediated, actions on endothelial cells, as the promotion of the expression and activity of eNOS [10,133]. Moreover, systemic or local infusions of GH acutely produces vasodilation in the forearm and NO delivery in healthy humans, without producing significant changes in plasma IGF-I levels or in muscle IGF-I mRNA expression. Preliminary data from our clinical trial in CLI confirm a significant increase of eNOS mRNA expression in ischemic muscle samples from CLI patients treated with GH as compared with those that were treated with placebo [193], without a significant increase of IGF-I levels neither in plasma nor skeletal muscle.

IGF-I may also mediate the proangiogenic actions of GH, as its receptors are expressed in endothelial cells, and it stimulates angiogenesis both in vivo and in vitro [131].

In line with these IGF-I effects, the local infusion of the IGF-I plasmid in skeletal muscle tissue, following ligation of the femoral artery in mice, leads to angiogenesis and raises the blood flow in the affected muscle [194]. Several studies indicate that IGF-I is a strong inducer of angiogenesis in different tissues, including the brain [195,196,197], protecting them from ischemia-induced apoptosis and inducing local expression of VEGF, as seen in cultures of ovarian granulosa cells [198].

Nonetheless, the systemic infusion of IGF-I causes negative collateral effects, such as hypoglycemia, hypotension, edema and tachycardia [199], the latter probably occurring as a result of the onset of hypoglycemia and/or hypotension. Furthermore, IGF-I is a mitogenic hormone with a marked oncogenic potential; hence, its long-term use in myocardial or arterial diseases should be avoided or carefully controlled. Although the hepatic (and that of many other tissues) expression of IGF-I is mainly dependent on GH, it does not take place if there is not an adequate liver metabolism of glucose [4]. Furthermore, since plasma levels of IGF-binding protein 3 (IGFBP3), which is a main carrier of IGF-I, are strongly dependent on GH, the bioavailability of IGF-I (free IGF-I), established by the IGF-I/IGFBP3 ratio, would not entail a major problem in the case of GH treatments, provided that it is well controlled.

As mentioned above, the angiogenic effects of GH do not only depend on its direct and IGF-I mediated actions on endothelial cells, or on EPC, but also on its indirect effects mediated by the induction of several growth factors such as VEGF, FGF, epidermal growth factor (EGF), BDNF, EPO, and some cytokines [81]. Besides, GH is capable of interacting with receptors for prolactin (PRL) which can trigger proangiogenic signals [200].

It is of interest the relationship between GH and C-X-C motif chemokine ligand 12 (CXCL12) or SDF-1, since both molecules orchestrate vasculogenesis, and share CXCR4 receptor present in pituitary somatotrophs [201]. Both also activate the JAK/STAT pathway, responsible for endothelial migration and differentiation during angiogenesis [201]. GH stimulates the secretion of SDF1 and the latter promotes GH delivery from the anterior pituitary gland [201,202,203].

**Figure 6:**
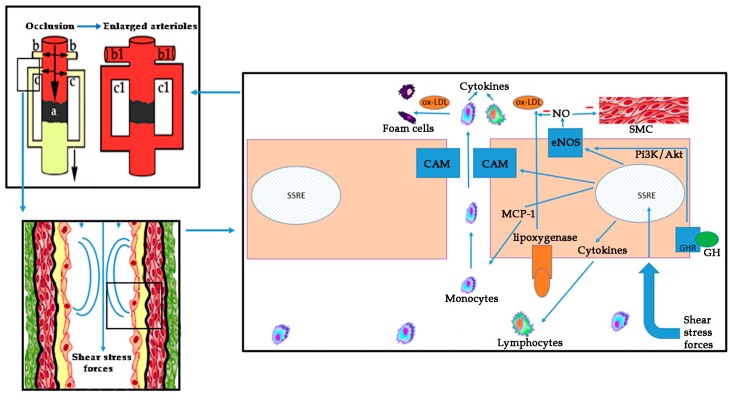
Mechanism of arteriogenesis. Oxidative stress at endothelial cell level produces LDL oxidation (ox-LDL) and foam cells formation that compose atheroma plaque, and, in the end, determine the vessels occlusion. After an arterial occlusion (a), the increase in shear stress forces through the collateral vessels (b, c) activates shear stress genes (SSRE) triggering the arteriogenic response. Adhesion molecules (CAM) and some cytokines such as MCP-1 are produced by the endothelial cells, attracting monocytes and lymphocytes from the blood to the vascular wall that start up the vascular remodeling (b1,c1). eNOs is also activated, increasing NO that produces vasodilation, inhibits SMC growing and the oxidation of LDL molecules. These facts lead to the control of the atheroma plaque, lowering oxidative stress. GH contributes to increase NO pathway by activating the Pi3K/Akt pathway. NO: nitric oxide; eNOS: endothelial nitric oxide synthase; ox-LDL: oxidized low density lipoprotein; GH: growth hormone; GHR: GH receptor; SSRE: shear stress response elements (promoter sequences that mediates the responsiveness of endothelial genes to shear stress); SMC: smooth muscle cells. Blue arrows indicate stimulation. Red line indicates inhibition of SMC growth and inhibition of ox-LDL. Black arrow indicates the absence of blood flow after the occlusion, due to the lack of enlargement of collateral arterioles.

GH may also enhance arteriogenesis. Collateral arteries enlargement is mediated by several pathways as Rho and RAS-ERK, both regulating cell proliferation and migration, respectively, and the NO pathway, which partially controls endothelial function and leukocytes adhesion [204]. The GH-IGF-I axis boosts the eNOS enzyme, increasing NO which, in parallel, inhibits the proliferation and migration of SMC and reduces platelet adhesion [152,205,206,207,208], leading to a control of the angiogenic process. Despite this, some models of PAD have no proven a real depletion of NO, but rather a marked insensitivity to the latter due to redox imbalance [209]. Figure 6 schematizes these concepts.

As a result of the described action of GH on the autonomic nervous system [210], there will be a removal of sympathetic constrictor tone from arterial walls, along with an increased blood flow in the denervated area, which may stimulate vessel enlargement, consequently leading to arteriogenesis.

The stimulation of VEGF by GH is another fact supporting the possible role of the hormone in arteriogenesis, since VEGF up-regulates CAMs, which are key factors for the development of collateral arteries [205].

MCP-1 and T-lymphocytes also mediate vascular remodeling during arteriogenesis [205]. GH strongly induces these cells [4,211,212]; for instance, it has been found that the exogenous administration of GH, there is a rise in *MCP-1* mRNA [211]. Moreover, would healing would benefit of the induction of immune system by GH. However, GHD in humans does not frequently associate with a significant affectation in immune system, as it happens in GHD animals [212], possibly because of the locally produced GH.

Both arteriogenesis and angiogenesis, are affected by age, mainly because of the alteration in EPC, and the parallel depletion in GH with aging has been related to the latter impaired processes. Physiological anti-angiogenesis is favored as we age, and a GH therapy for elderly people has been proposed to compensate this imbalance. Apart from its metabolic effects, a therapy with this hormone would improve the eNOS system dysfunction with aging, and, hence, the arteriogenic and angiogenic mechanisms. If we consider that most patients suffering from PAD are of old age, this therapy could be justified, at least as a complementary treatment, as long as it has been ruled out that no significant contraindications, such as a severe illness, sepsis or a tumor, among others. Maybe it would be necessary to define the most profitable type of therapy with the hormone. In any case, GH treatment does not need to last long time for showing significant benefits. Moreover, they can be interrupted during some months and resumed later. Once again, physiological level of GH or short-term GH excess, have positive effects, whereas long-term GH excess would be negative.

### 2.7. Effects of GH on Nerve Dysfunction and Other Abnormalities in the Ischemic Muscle

Ischemia also damages lower extremity nerves in PAD patients, causing functional impairment. The observation of muscle denervation in these patients supports the idea that arterial flow insufficiency coexists with distal motor neuron neuropathy that worsens muscle function [213]. However, cross-sectional studies have shown conflicting results, which is probably due to small sample sizes. WALCS II study has found an ABI-dependent effect in non-diabetic PAD patients on the nerves. Patients with ABI < 0.5 was associated with poorer peroneal nerve conduction velocity. For those being diabetic, the injury to the nerves was simply related to the fact of suffering or not from PAD [99].

In this sense, the actions of GH treatment on neurogenesis and peripheral nerve recovery after an injury have been described [214]. Therefore, GH administration may also be useful in the nerve affectation of PAD patients.

However, patients with PAD show many other changes in their lower extremities skeletal muscles, among them: muscle apoptosis and atrophy, increased fiber type switching, altered myosin heavy-chain expression, and muscle fiber denervation [215,216]. All of these changes impair exercise tolerance and performance, and could be produced by the greater inflammatory response that these patients present [99]. In samples from the gastrocnemius muscle of PAD patients, caspase-3 levels are twice as high as in control patients [216]. The role of GH in increasing the skeletal muscle mass has been broadly described. The GH-IGF-I axis constitutes an important physiological regulatory mechanism for coordinating postnatal skeletal muscle expansion and hypertrophy. The administration of GH to both animals and GHD humans improves muscle strength [217,218]. When considering that most of patients with PAD are aged people with a physiologic GH deficiency, and that sarcopenia appears along aging, GH therapy might be useful for recovering muscle mass and performance [4]. GH activates the IGF-I-Akt-mTOR pathway in the skeletal muscle, which mediates both differentiation in myoblasts and hypertrophy in myotubes, and inhibits myostatin-dependent signaling [219,220]. Thereby, in this regard, GH could also help in the recovery of PAD patients.

### 2.8. Why GH Treatment Could not Work Properly in Cardiovascular Disease?

With independence of all described GH vascular actions, the final vascular effects will depend on the local environment in which the hormone tries to act. In this sense, high levels of GH can be seen without angiogenesis stimulation. In fact, some harvested endothelial cells does not proliferate with GH [200]. The presence of other angiogenic agents, such as IGF-I, NO, VEGF, and even the autocrine GH itself may determine the final effect of the exogenous GH. The autocrine hormone could saturate the GHR, thus avoiding the action of endocrine or exogenous GH. Additionally, when GH is internalized, it may suffer a proteolytic cleavage, generating vasoinhibins, being then inactivated. This helps to balance growth and regression of blood vessels under physiological conditions, especially in the female reproductive system. The role of vasoinhibins has been reviewed in detail [200,221].

The suppressor of cytokine signaling (SOCS) family is also involved in the regulation of GH signaling. This relationship is not still well understood, since, for example, high levels of SOCS2 up-regulate GH signaling, while less concentrations of the cytokine inhibits GH activity [222]. In PAD patients, pro-inflammatory IL-1β or TNF-α, and endotoxins, are frequently increased as a consequence of the inflammation and infection at the level of the foot, and these cytokines may stimulate SOCS proteins, promoting a GH insensibility.

### 2.9. Adverse Effects of GH Treatment

**Figure 7:**
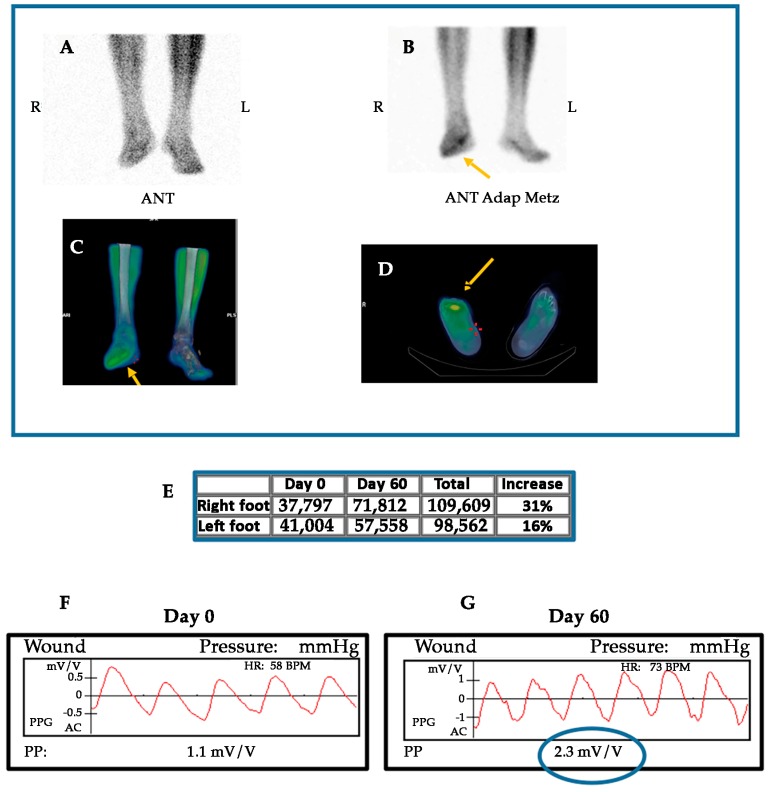
Combination of gammagraphy with leucocytes marked with technetium 99 m (**A**,**B**) and single photon emission computed tomography (SPECT)-MIBI (**C**,**D**), showing the increasing of flow in the right foot of a diabetic patient suffering from PAD (critical ischemia of the right limb). Before the treatment ((**A**); Day 0), and after two months of GH treatment ((**B**–**D**); Day 60). Arrows indicate the blood flow. The table shows the quantification of ROI (region of interest), demonstrating a final increase of flow of 31% of the right foot as compared with the same foot at day 0 (**E**). Photoplethysmography (PPG) from the same patient, showing the improvement in microcirculation at the wound level between the day 0 and (**F**) and the day 60 (**G**) after GH treatment. Blue circle depicts the increase in pulsatility (PP), measured in mV/V. HR: heart rate.

Although GH opposes to the effects of insulin and might produce hyperglycemia and diabetes, this is not a clear contraindication for the possible use of the hormone in the case of diabetic patients with PAD. The incidence of diabetes in the GHD population treated with the hormone is very low. Data from more than 23.333 young people aged 10-19 treated with GH for growth disorders for a mean of two years showed an incidence of 46.3/100,000/year of GH therapy of type 2 diabetes. In old people and children, it is even less [223]. Although some physicians remain unsure about using GH for this reason and because the incidence of abnormal glucose tolerance is higher, this is not a strong argument to contraindicate the hormone therapy in diabetic patients, as a recent study reported that GH and its receptor regulate the pancreatic β-cell survival and insulin secretion in rats [224]. GH corrects insulin sensitivity and long-term glycemic control without altering glycated haemoglobin (HbA1C) levels [223,225]. In addition, the plasma half-life of GH is very short (approximately 30 min) and the slightly increased glycemia that the hormone produces, may be minimized by exercise, since the working muscle has a great avidity to capture glucose in an insulin-independent manner. In fact, in the GHAS study, with a 70% prevalence of diabetic patients, we did not find significant differences in plasma levels of glucose or HbA1C between patients treated with GH or placebo, from baseline to the end of the study. This supports the fact that the treatment with GH during short-time periods does not produce glycemic alterations and may be administrated in diabetic patients with PAD, improving their peripheral ischemic problem, as Figure 7 shows.

Concern arises with the oncogenic potential of GH. First, this is something inherent to most of the growth factors that are used for neovascularization. Second, long-term studies in GHD children do not show any increase in the incidence of tumors; moreover, the slight increase in the incidence of a second tumor detected in children with GHD secondary to leukemia treated with associated prophylactic brain radiotherapy, has been shown to be a consequence of the radiotherapy and not of the treatment with GH. Third, although an increased risk of thyroid cancer in acromegaly has been published [226], this condition implies a very high and sustained release of GH for many years, and, therefore, we cannot be sure of the fact that it is GH and no other factors (for example, IGF-I) responsible for the slight increase in prevalence of cancer in patients with acromegaly. On the other hand, the association between GH and tumors (breast, colon, prostate, etc.) has mainly been established because of the detection of the hormone and its receptor in tumor cells. However, it is necessary to remind that GH is produced in almost any tissue and organ, and therefore, this auto/paracrine GH might be responsible of the development of a tumor in susceptible patients. In our own experience, short-term treatment with a strict control of plasma IGF-I levels is safe and effective. In any case, it is necessary to rule out a neoplasm if GH therapy will be scheduled, especially in the elderly population.

Additionally, it has been found that acromegaly also raises the risk of atherosclerosis [227,228,229]. This argument is contrary to the fact that GH is an atherosclerotic protector, but it must be elucidated whether the latter is a consequence of the increase in cardiovascular risk factors, rather than a pure consequence of the hormone.

Another adverse effect of the hormone is the development of carpal tunnel syndrome, but this is unlikely to occur during short-time treatments with GH as these that we propose for treating CVD and PAD.

### 2.10. Controversies Regarding GH Treatments

In 2012, a very interesting study [230] compared the effects of GH excess on the cardiovascular system in two very different situations, acromegaly and doping in a series of athletes practicing baseball, cycling, and in track and field, concluding that there are adverse cardiovascular outcomes in both so different situations.

The case of acromegaly has already been described here. It is a consequence of a maintained uncontrolled hypersecretion of GH during years, which evolves in silence until the first clear symptoms appear around 10 years after the beginning of the pituitary tumor process. In this disease, there is an early hyperkynetic syndrome with an accelerated heart rate, which is followed by diastolic dysfunction and consequently affected LV filling. Further, peripheral vasculature is affected with impaired endothelium-dependent vasodilation, and the IMT is significantly increased.

The other situation, doping with GH lacks physiological coherence. In our opinion, GH excess, apart of its adverse effects on the heart, would mainly lead to a dangerous increase of the hematocrit, which while being good for increasing O_2_ supply, also might lead to the development of thromboembolism.

Short-term GH administration to healthy subjects, only reported conflicting data, because in terms of heart rate, while an increase as small as 5 beats/min was communicated, other studies did not find significant changes in heart rate after GH treatment. However, studies in healthy rats showed that when they are treated with a GH excess a cardiac hyperthropy exists [231]. In any case, athletes utilizing GH for doping, use it at doses quite higher that those used for treating GHD; moreover, they usually receive different types of anabolic steroids. Therefore, this type of population seems not to be the ideal to analyze the physiological effects of GH on the heart.

In 2014, it was published an intriguing report [232], in which it was described that a novel high sensitive (hs) GH assay (detecting pg/mL, instead of the usual ng/mL of standard assays) correlated the number of hs-GH peaks with high cardiovascular morbidity and mortality (CAD, Stroke, Congestive Heart Failure) in a high population of Swedish patients. The study has not been replicated yet, but the magnitude of the association was quite discrete, therefore needing further and larger studies [233]. Moreover, plasma samples were withdrawn between 7.30 and 9.00 a.m., a period of time in which, usually GH secretion is absent [234]. In his work the authors did not describe the frequency of sampling, a factor that can have an influence in the results obtained, or the mathematical method for establishing when a GH pulse appears (such as deconvolution methods). Moreover, the possibility exists that high hs-GH peaks may have a role on inducing IGF-I or Insulin release, responsible for the increased CVD found in these patients. In any case, it is an interesting study that merits further and more complete investigations.

### 2.11. Future Perspectives

From the concepts described in this review, supported by our GHAS study and previous data from other group [21], it is likely that a short-time GH treatment may be a good alternative for cardiovascular diseases. In fact, eight years ago, one of us (JD) treated with GH (0.4 mg/day) a 72-year-old man who had suffered an ischemic stroke 17 years earlier, whose sequelae was a spastic hemiplegia that had forced him to remain in a wheelchair. Over the years, he developed a critical ischemia of his limbs; an echo-Doppler revealed bilateral atheromatosis. In the lower right limb, there was decreased flow in the tibio-fibular trunk, while in in the left limb, there was superficial femoral occlusion from its origin and complete absence of flow in tibio-peroneal trunks. These led him to be programmed for the amputation of his left leg, because gangrene began to develop in his left foot. However, before surgery was carried out we began to treat him (after obtaining signed informed consent) with GH, and soon the situation began to improve and amputation was discarded.

The patient was treated for three months, followed by one-month resting, and after this, another three months GH was given. Eight years later he still retains his leg, there are no sequels and several vascular studies indicate that the blood flow in his affected leg is conserved at 80 years of age.

A logical future goes through the transfection by means of viral vectors of factors involved in vascular or myocardial regeneration, or the transplantation of CD34+ cells into ventricular tissue damaged by myocardial injury.

In the case of stem cells transplants, there are studies indicating that intramyocardial injection of Mesenchimal Stem Cells overexpressing the survival factor Akt may significantly repair infarcted myocardium in rats and improve cardiac function, as early as three days after the injection, despite that only a small number of MSCs differentiated into cardiomyocytes [235]. It seems to be clear that in addition of the survival role that Akt plays [135], cytokines and growth factors released by the implanted MSCs contribute to the results that were obtained in that study [235]. In addition, timing of intracoronary transplantation in acute myocardial infarction is another key factor for positive outcomes, as a recent meta-analysis of 34 randomized controlled trials shows [236]. Curiously, the ideal window of time for this therapy ranges from three to seven days, rather than within 24 h after the acute myocardial infarction, as one would think. Another key factor for positive outcomes is the number of MSCs administered, no lesser than 10^8^–10^9^ [237].

Despite these promising previous studies, the current situation is still far from being clear. A number of preclinical and clinical studies have analyzed the potential of endothelial progenitor cells (EPC) and cardiac stem cells (CSC) for repairing cardiovascular diseases, but while some of these studies show improvements in left ventricular ejection fraction in patients with acute myocardial infarction, other results have been poor or no significant clinical benefit has been observed in many cases [238].

Another source of stem cells, possibly useful for being used after a cardiac infarction, is the adipose tissue surrounding the heart. From this tissue MSCs can be isolated, and when injected intramyocardially in postinfarcted mice and rats enhance myocardial vascularization, reduce the infarct size, and express cardiac and endothelial markers [239]. However, these stem cells have not yet been tested in clinical studies [240].

In any case, the parallel administration of GH would facilitate the proliferation and survival of these cells, as we observed in brain injuries [241].

Regarding to the viral-vector delivery of growth factors, there are encouraging results obtained in many animal models of lower extremity ischemia, in which an improvement in the perfusion of hypoxic tissues has been described using viral-vector delivery growth factors (mainly FGF-2 and VEGF) [242], However, the results in human patients with CLI did not reveal significant benefits when using this type of therapies. Moreover, this type of therapy is not free of adverse effects [243,244,245].

An important reason explaining why VEGF has failed on clinical trials, may be due to that when VEGF is administered, it induces the formation of new vessels in a chaotic, unstable and not functional way. New vessels need of a lumen to be functional, but VEGF alone is insufficient for the lumenogenesis process. In this process, the activation of the protein kinase Akt and R-Ras seems to be the key [246]. That is, Akt1-deficiency compromises endothelial cells sprouting, and impairs the morphogenesis of the new vessels, making these vessels inadequate for re-oxygenating the ischemic tissues [247]. VEGF activates Akt to induce the sprouting of endothelial cells. Once this phenomenon has been initiated, R-Ras stimulates Akt to induce lumenogenesis. In this second step, the microtubule cytoskeleton is stabilized in the migrated endothelial cells, creating a stable structure and the lumen [248]. The latter is essential for nutrients and oxygen transport and delivery to ischemic tissues. Microtubule network is essential for endothelial cells polarization, lumenization, and stabilization of the endothelial lumen structure [249]. On one side, GTPase protein R-Ras activates Pi3K/Akt signaling [250], facilitating the maturation of vessels in the new vasculature [251], and the association of pericytes with nascent blood vessels. On the other hand, it inhibits an excessive sprouting and branching of angiogenic vessels to control the process. Again, it is necessary to remark that both GH and IGF-I are powerful inductors of the Pi3K/Akt pathway, and, from this point of view, might aid VEGF in its neovascularization actions. Thus, the combination of both, VEGF and GH, could be advisable and tested to increase the possibilities of improving the clinical endpoints in trials dealing with angiogenic molecules in cardiovascular diseases. That is, while only one factor may be capable of starting up the angiogenic process, there may be also the need for other cytokines to set it up [252,253,254]. For example, in animal models of ischemia, the association between granulocyte colony-stimulating factor (GCSF) and mononuclear cells induced a greater revascularization phenomenon than the administration of both molecules separately [252]. The same happened with the combination of VEGF and FGF, with a greater and more rapid increase in collateral circulation [255]. Because of the possibility of collateral effects, a short-term period of association of several growth factors might be more suitable to avoid oncogenic potential.

In addition, some studies have described that, rather than a lack of growth factors in patients with PAD, there is an altered response to them. As we stated above, not all of the studies agree, since in the elderly population it seems to be a real depletion of growth factors, but the differences between VEGF receptor flt-1 could explain the inter-subject differences with respect to the VEGF effect. Apart from those mentioned above, another problem related to human ischemia is the existence of a real endothelial dysfunction. This also impairs the arteriogenic response, which needs to be the ultimate goal in PAD patients.

Vasodilation of collateral vessels and a decreased resistance to blood flow occurs because of arteriogenesis. Paradoxically, in leg ischemia oxygen tension drops in the foot, whilst a collateral network is generated in the thigh, where they are adequately oxygenated tissues. Therefore, although therapeutic interventions aimed at salvaging a limb affected by critical ischemia may include attempts to stimulate both angiogenesis and arteriogenesis, the latter should be predominant if we seek to find a real clinical benefit in the standard patient with a major artery occlusion. Taken together, all of these facts explain why different growth factors that are used in PAD have failed in the target: there are not mature angiogenic vessels, there is a lack of stimulation of arteriogenesis, and a lack of correction of the main process involved in the pathogenesis of this problem, the endothelial dysfunction. This is corrected by GH administration, independently of the patient is GHD or not.

## 3. Conclusions

From this review and our own data, we can conclude that short-time GH administration may be useful for improving the endothelial dysfunction, leading to the development of atherosclerosis, or correcting it when it is established. GH improves the redox imbalance, enhances the appearance of collateral vessels after a major artery occlusion, improves wound healing, and ameliorates cardiac functioning after a myocardial infarction or heart failure. Short-term GH treatment can be safely given to elderly patients suffering cardiovascular diseases, regardless of whether they are GHD or not, whenever a severe contraindication does not exist. Although the association of GH and other growth factor might be advisable, short-term association is also recommendable to avoid the oncogenic potential and collateral effects. The relationships between Klotho and GH merit further studies, but the possibility exists that Klotho might be released from the dysfunctional endothelium to induce a secretion of GH that would be able to repair this damaged tissue.
